# Two Marine *Desulfotomaculum* spp. of Different Origin are Capable of Utilizing Acetone and Higher Ketones

**DOI:** 10.1007/s00284-021-02441-9

**Published:** 2021-03-22

**Authors:** Jasmin Frey, Sophie Kaßner, Bernhard Schink

**Affiliations:** Department of Biology, University of Konstanz, 78457 Konstanz, Germany

## Abstract

Degradation of acetone and higher ketones has been described in detail for aerobic and nitrate-reducing bacteria. Among sulfate-reducing bacteria, degradation of acetone and other ketones is still an uncommon ability and has not been understood completely yet. In the present work, we show that *Desulfotomaculum arcticum* and *Desulfotomaculum geothermicum* are able to degrade acetone and butanone. Total proteomics of cell-free extracts of both organisms indicated an involvement of a thiamine diphosphate-dependent enzyme, a B_12_-dependent mutase, and a specific dehydrogenase during acetone degradation. Similar enzymes were recently described to be involved in acetone degradation by *Desulfococcus biacutus*. As there are so far only two described sulfate reducers able to degrade acetone, *D. arcticum* and *D. geothermicum* represent two further species with this capacity. All these bacteria appear to degrade acetone via the same set of enzymes and therefore via the same pathway.

## Introduction

Acetone is a common pollutant in nature and originates either from anthropogenic sources like industrial wastewater and oil spills, or from natural production by some solventogenic *Clostridium* species [1–3]. In seawater, acetone is formed by photochemical processes [4]. Several pathways of acetone degradation have been described for aerobic and nitrate-reducing bacteria. While aerobes metabolize acetone either via oxygenases to acetol, or to methyl acetate using an O_2_-dependent Baeyer–Villiger monooxygenase, or by an ATP-dependent carboxylation to acetoacetate [5–8], anaerobic bacteria (phototrophic and nitrate-reducing ones) activate acetone through carboxylation to acetoacetate [9–11].

Only little is known about acetone degradation by sulfate-reducing bacteria. Two sulfate-reducing strains were described which utilize acetone as carbon source: *Desulfococcus biacutus* strain KMRSAct and *Desulfosarcina cetonica* strain 480 [12, 13]. Genomic and proteomic studies with *Desulfococcus biacutus* identified a gene cluster that codes for proteins which were specifically induced during growth with acetone. One of these proteins was annotated as a thiamine diphosphate (TDP)-requiring enzyme, two others were annotated as two subunits of a B_12_-dependent mutase and a specific dehydrogenase [14, 15]. A similar gene cluster was identified in *Desulfosarcina cetonica*, *Desulfotomaculum arcticum,* and *Desulfotomaculum geothermicum* [15].

The present study was designed to elucidate the ability of *D. arcticum* and *D. geothermicum* to utilize acetone as sole carbon source for growth. Growth experiments were performed with *D. arcticum* growing with acetone, butanone, and isopropanol in comparison to butyrate as a control. Butanone and isopropanol served as substrates related to acetone. Additionally, proteomic data were obtained for *D. arcticum* and *D. geothermicum* to investigate specifically acetone-induced protein production.

## Methods

### Chemicals

All chemicals were purchased from Sigma-Aldrich (Germany), AppliChem (Germany) or Carl Roth GmbH (Germany) and were at least of analytical grade.

### Bacterial Growth Conditions

*Desulfotomaculum arcticum* strain 15 (DSM 17038) and *D. geothermicum* strain BSD (DSM 3669) were cultivated in N_2_/CO_2_ (80/20)-flushed, rubber-stoppered flasks containing sulfide-reduced, bicarbonate-buffered marine mineral medium with trace element solution SL-10 and a 10-vitamin solution (containing µg/L medium: cyanocobalamin (B_12_) 1; *p*-amino benzoic acid 50; biotin 20; Ca-D(+) pantothenate 50; pyridoxine (B_6_) 100; thiamine (B_1_) 50; folic acid 20; riboflavin (B_2_) 50, lipoic acid 50 and nicotinic acid 50) (modified after [16]). The medium was usually supplemented with 10 mM Na_2_SO_4_ as electron acceptor and 5 mM carbon source (acetone, butyrate, isopropanol or butanone); for determination of growth parameters, excess Na_2_SO_4_ (10–15 mM) was used. Cultures were incubated in the dark at 37 °C (*D. arcticum*) or 50 °C (*D. geothermicum*), corresponding to the reported optimal growth temperatures of these strains.

### Preparation of Cell-Free Extracts

Cells of *D. arcticum* and *D. geothermicum* were harvested by centrifugation (8200×*g*, 30 min, 4 °C) and washed two times with Tris–HCl buffer (20 mM, pH 7.2). The cell pellet was resuspended in Tris–HCl buffer (20 mM, pH 7.2) supplemented with 0.5 mg DNase mL^−1^ and 10 µL mL^−1^ of Halt™ Protease Inhibitor Cocktail (with EDTA; Thermo Scientific). Cells were disrupted by three to five passages through a cooled French pressure cell (140 MPa). Cell debris was removed by centrifugation (27,000×*g*, 30 min, 4 °C) to obtain cell-free extract.

### Protein Analysis

For total proteome analysis of cell-free extracts (CFE) of cells grown with different substrates (acetone, butyrate, isopropanol, and butanone), samples of CFE were analyzed with high-resolution (Orbitrap) peptide fingerprinting-mass spectrometry by the Proteomics Facility of University of Konstanz. Samples were digested with trypsin and were then analyzed by liquid chromatography nanospray tandem mass spectrometry (LC–MS/MS). A LTQ-Orbitrap mass spectrometer (Thermo Fisher) in combination with an Eksigent nano-HPLC were used with a reversed-phase LC column (5 mm, 100 Å pore size C18 resin in a 75 mm i.d. × 15 cm long piece of fused silica capillary, Acclaim PepMap100, Thermo Scientific). After injection of the sample, a washing step with 10% of eluent B (0.1% formic acid in acetonitrile) and 90% of eluent A (0.1% formic acid) was applied for 5 min with a flow rate of 300 nl/min. Afterwards, a linear gradient of 10% to 35% of eluent B in 95 min was applied to elute the peptides, followed by a washing step of 5 min (35 to 80% eluent B). The data-dependent mode was used for operating the LTQ-Orbitrap mass spectrometer. A protein database (obtained from the Joint Genome Institute (JGI IMG)) of *D. arcticum* or *D. geothermicum* was searched against tandem mass spectra using Mascot (Matrix Science). For semi-quantitative analysis of relative protein abundance, the area values of the respective peaks in the ion chromatogram were analyzed using the Proteome Discoverer software (Thermo Fisher).

### Analysis of Side Products via High-Pressure Liquid Chromatography (HPLC)

Potential side products like acetate and propionate were analyzed using HPLC using a method that was previously described [17]. A Shimadzu system with an RID detector (RID-10A, Shimadzu, Japan) was used, employing an isocratic method with 5 mM H_2_SO_4_ as eluent at a flow rate of 0.6 ml per min. Compounds were separated on a RezexTM.

RHM-Monosaccharide HC (8%) ion exchange resin column (LC column 300 mm × 7.8 mm, 00H-0132-K0, Phenomenex, Los Angeles, United States) at 60 °C.

## Results and Discussion

### Growth with Acetone, Butyrate, Butanone and Isopropanol

DNA sequence analysis of *D. arcticum* and *D. geothermicum* indicated that both contain a gene cluster that was proposed to be crucial for acetone degradation [15]. Therefore, these two strains were cultivated with acetone (as well as with butanone or isopropanol as compounds related to acetone) as sole carbon source, to check whether possession of these genes enables microorganisms to grow with the respective substrates.

Both strains grew with butyrate or acetone as sole carbon source, *D. arcticum* grew also with isopropanol or butanone. *D. geothermicum* utilized butanone but not isopropanol. No side products such as acetate or propionate (for growth with butanone) were detectable during growth with acetone, isopropanol (only *D. arcticum*) or butanone. As *D. geothermicum* grew to lower cell densities than *D. arcticum*, only *D. arcticum* was used for further growth experiments and calculations. In Table 1, cell mass formation, substrate consumption, product formation and electron recovery of *D. arcticum* under all four growth conditions are shown.

**Table 1:** Growth of *D. arcticum* with different substrates

	∆ OD	substrate degraded (mM)	Cell dry mass formed (mg)^b^	Substrate assimilated (mM)^c^	Substrate dissimilated (mM)	Sulfide formed (mM)	substrate consumed calc. via sulfide formation^d^	Electron recovery (%)
Acetone	0.12	4.89^a^	29.00	0.30	4.59	9.17	4.58	93.79
Butyrate	0.18	4.72	45.58	0.52	4.34	11.47	4.59	97.22
Isopropanol	0.16	4.47^a^	39.25	0.36	4.11	10.12	4.50	100.69
Butanone	0.18	5^a^	43.75	0.33	4.67	9.23	3.36	67.12

All cultures were analyzed in biological triplicates

^a^Calculated from 5 mM amended substrate

^b^Calculated with an experimentally determined value of 250 mg cell dry mass per liter culture with OD_600_ of 1

^c^Assimilation of substrate was calculated according to the following equations (based on the theoretical formula for cell mass [C_4_H_7_O_3_]: Acetone: 17 C_3_H_6_O + 13 CO_2_ + 5 H_2_O → 16 [C_4_H_7_O_3_]; Butyrate: 17 C_4_H_8_O_2_ + 12 CO_2_ + 2 H_2_O → 20 [C_4_H_7_O_3_]; Isopropanol: 17 C_3_H_8_O + 21 CO_2_ → 18 [C_4_H_7_O_3_] + 5 H_2_O; Butanone: 17 C_4_H_8_O + 20 CO_2_ + 9 H_2_O → 22 [C_4_H_7_O_3_]

^d^Calculation according to the following equations: Acetone: C_3_H_6_O + 2 SO_4_^2−^ + 2 H^+^  → 3 CO_2_ + 3 H_2_O + 2 H_2_S (= 2 SO_4_^2−^/Act); Butyrate: 2 C_4_H_8_O_2_ + 5 SO_4_^2−^ + 10 H^+^  → 8 CO_2_ + 8 H_2_O + 5 H_2_S (= 2.5 SO_4_^2−^/But); Isopropanol: 4 C_3_H_8_O + 9 SO_4_^2−^ + 18 H^+^  → 12 CO_2_ + 16 H_2_O + 9 H_2_S (= 2.25 SO_4_^2−^/Ipr);; Butanone: 4 C_4_H_8_O + 11 SO_4_^2−^ + 22 H^+^  → 16 CO_2_ + 11 H_2_S (= 2.75 SO_4_^2−^/Bnon)

*Desulfotomaculum arcticum* and *Desulfotomaculum geothermicum* both were originally described as sulfate-reducing bacteria capable of complete substrate oxidation, forming CO_2_ and H_2_S as the only end products. Both strains were reported to grow with a variety of short-chain acids like lactate, propionate and butyrate. Whereas *D. geothermicum* has been described to utilize ethanol as the only short-chain alcohol, *D. arcticum* utilizes methanol, ethanol, propanol and butanol [18, 19]. Utilization of ketones and secondary alcohols has not been reported for these sulfate reducers so far.

### Substrate-Specific Enzyme Induction in *D. arcticum*

**Fig. 1:**
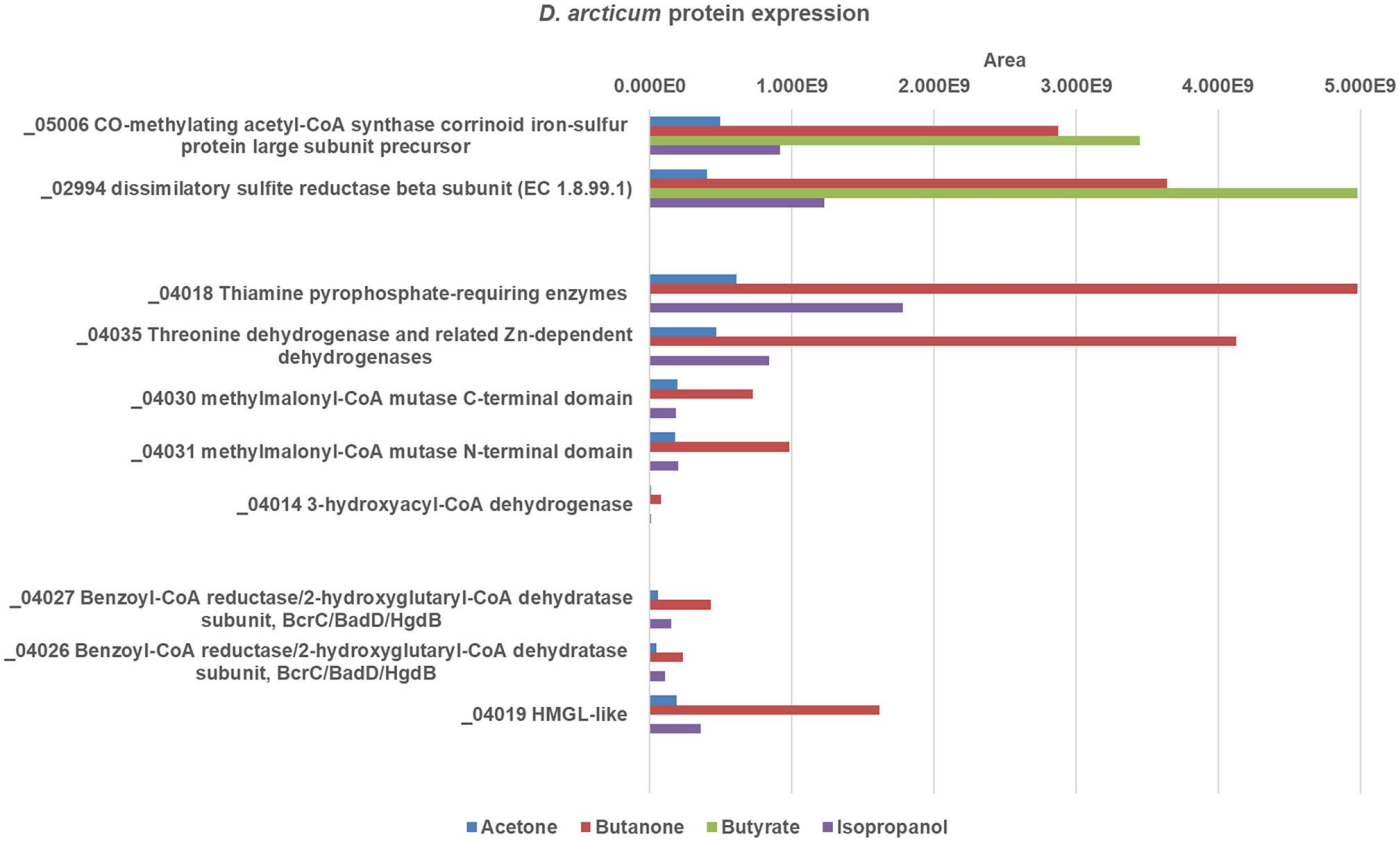
Bar plots depicting expression of enzyme proteins produced by *D. arcticum* after growth with different substrates*.* Growth substrates were: red: butanone, green: butyrate, purple: isopropanol, blue: acetone; Area values refer to relative protein abundance (color figure online)

Using proteomics with cell-free extracts (CFE) of *D. arcticum,* several specifically acetone-induced proteins were identified. A thiamine diphosphate (TDP)-requiring enzyme [IMG locus tag: Ga0056061_04018; in the following the prefix Ga0056061_ is omitted)], a threonine dehydrogenase (04035), two subunits of a B_12_-dependent methylmalonyl-CoA mutase (04030, 04031) and a 3-hydroxyacyl-CoA dehydrogenase (04014) were highly abundant in extracts of acetone-grown cells, compared to butyrate-grown cells (see Fig. 1). Compared to the respective protein of *D. biacutus* [using the NCBI Basic Local Alignment Search Tool for proteins (Protein BLAST)], the TDP-requiring enzyme exhibits an identity of 55.83% at the amino acid level. The small subunit of the B_12_-dependent enzyme shows 48.46% identity, the large subunit 46.78%, and the above-mentioned 3-hydroxyacyl-CoA dehydrogenase has an identity of 31.94%. However, if the amino acid sequences of the respective proteins from *D. arcticum* are compared to those of *D. geothermicum*, identities are much higher: the TDP-dependent enzyme is 88.76% identical, the small subunit of the mutase shows an identity of 73.88%, the large subunit 84.35% and the dehydrogenase 57.78% in comparison to the respective proteins in *D. geothermicum*.

**Fig. 2:**
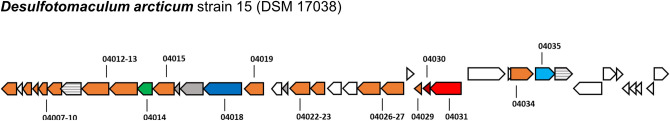
Gene cluster of *D. arcticum* containing genes coding for proteins relevant for acetone degradation. Color-coded genes are all highly abundant in acetone-, butanone-, and isopropanol-grown CFE in comparison to butyrate-grown CFE. Dark blue: acetolactate synthase (04018); red: small and large subunit of a methylmalonyl-CoA mutase (04030, 04031); green: 3-hydroxyacyl-CoA dehydrogenase (4014); light blue: threonine dehydrogenase (04035); orange: sodium ion-translocating decarboxylase (04005), carbonic anhydrases/acetyltransferases (04007), 4Fe-4S dicluster domain (04008), 2-oxoacid:acceptor oxidoreductase (04009), pyruvate:ferredoxin oxidoreductase (04010), acetyl-CoA carboxylase (04012), acyl-CoA synthetase (AMP-forming) (04013), acetyl-CoA acetyltransferase (04015), HMGL-like (04019), acetyl-CoA acetyltransferase (04022), enoyl-CoA hydratase/carnitine racemase (04023), two benzoyl-CoA reductase/2-hydroxyglutaryl-CoA dehydratase subunits (04026, 04027), 3-hydroxybutyryl-CoA dehydratase (04029), Xaa-Pro aminopeptidase (04034). Gray-labeled genes are expressed in specific conditions: hypothetical protein (04016; in acetone and butanone only), pyruvate-formate lyase-activating enzyme (04017, in acetone only), DUF35 OB-fold domain/rubredoxin-like zinc ribbon domain (04021, in acetone and isopropanol only); Gray-striped genes are constitutively expressed under all conditions; white-labeled genes were not detected in proteome analysis (color figure online)

Moreover, a HMGL-(hydroxymethylglutaryl-CoA lyase)-like protein (04019), two subunits of a benzoyl-CoA reductase/2-hydroxyglutaryl-CoA dehydratase (04026, 04027) as well as proteins annotated as a sodium ion-translocating decarboxylase (04005), a carbonic anhydrase/acetyltransferase (04007), a 4Fe-4S dicluster domain (04008), a 2-oxoacid:acceptor oxidoreductase (04009), a pyruvate:ferredoxin oxidoreductase (04010), an acetyl-CoA carboxylase, carboxyltransferase component (04012), an acyl-CoA synthetase (AMP-forming) (04013) and an acetyl-CoA acetyltransferase (EC 2.3.1.9) (04015) were also found to be induced in CFE of acetone-grown cells as compared to control cultures grown with butyrate. The genes coding for the respective proteins are all located in one gene cluster (see Fig. 2). In this gene cluster several more proteins were identified to be highly abundant during growth with acetone: a 3-hydroxybutyryl-CoA dehydratase (04029), a Xaa-Pro aminopeptidase (04034), an acetyl-CoA acetyltransferase (04022) and an enoyl-CoA hydratase/carnitine racemase (04023).

All of the above-mentioned acetone-induced proteins were also highly abundant in extracts of cells grown with butanone or isopropanol as compared to control cultures grown with butyrate. This implies that the enzymes of the acetone degradation pathway are used for degradation of butanone and isopropanol as well.

### Acetone-Specific Protein Induction in *D. geothermicum*

**Fig. 3:**
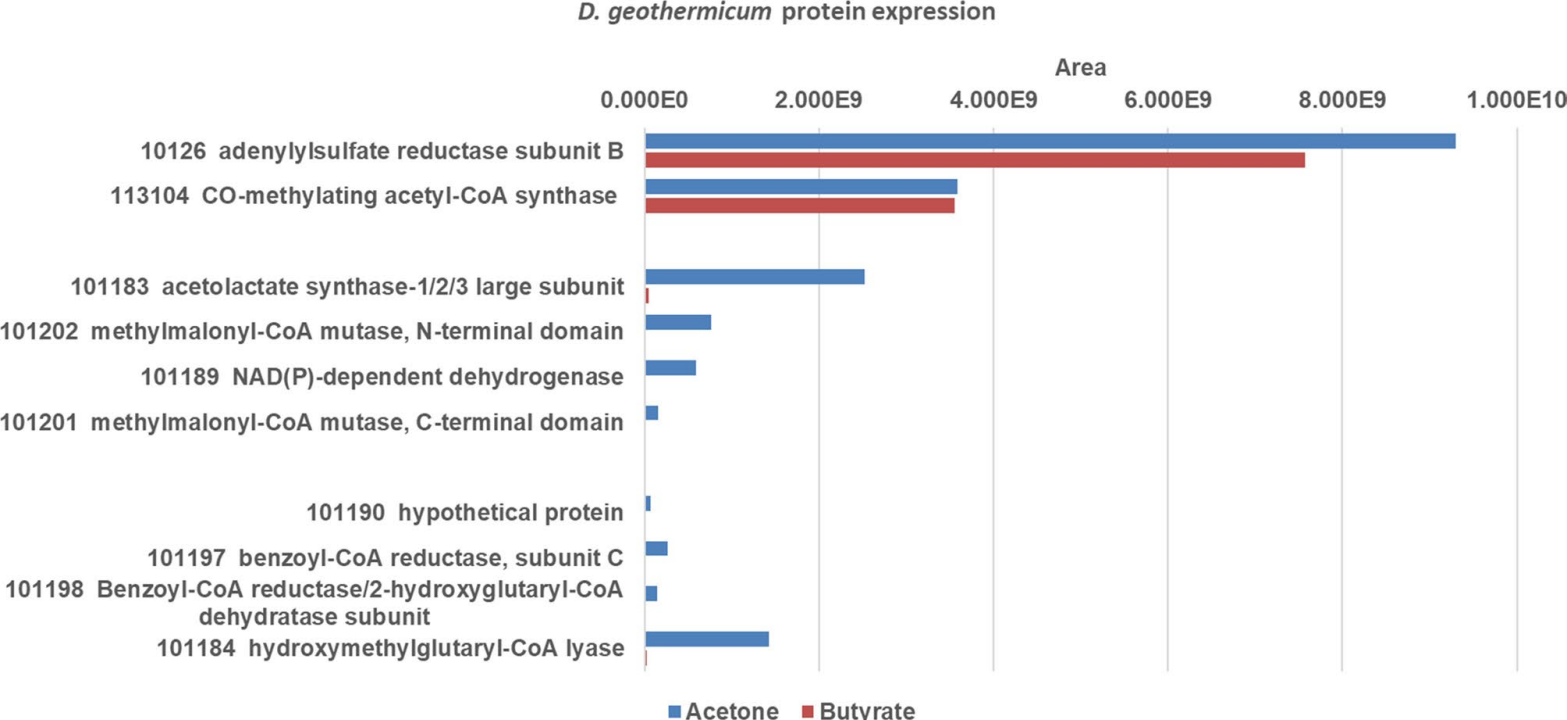
Bar plots depicting expression of enzyme proteins by *D. geothermicum* after growth with different substrates. Growth substrates were: red: butyrate, blue: acetone; Area values refer to relative protein abundance (color figure online)

Acetone-induced protein expression was investigated by comparison of expression patterns of cell-free extracts of acetone-grown and butyrate-grown cells of *D. geothermicum* using proteomic analyses (see Fig. 3).

Highly induced by acetone was a protein annotated as acetolactate synthase-1/2/3 large subunit (IMG locus tag: Ga0056068_101183; in the following text the prefix Ga0056068_ is omitted), which is a thiamine diphosphate (TDP)-dependent enzyme. Also two subunits (SU) of a B_12_-dependent mutase (annotated as methylmalonyl-CoA mutase; large SU: 101202, small SU: 101201) and a NAD(P)-dependent dehydrogenase of the short-chain alcohol dehydrogenase family (101189) were specifically induced after growth with acetone.

Comparison of these four proteins with the respective proteins of *D. biacutus* (using NCBI protein BLAST) showed an identity (at the amino acid level) of 55.24% for the TDP-dependent enzyme, 46.62% for the small SU, 46.88% for the large SU of the B_12_-dependent mutase, and 34.57% for the dehydrogenase.

Furthermore, several other proteins were found to be induced during growth with acetone. A very high specific abundance compared to butyrate-grown cells was observed for a hydroxymethylglutaryl-CoA lyase (101184) and for two subunits of a benzoyl-CoA reductase/2-hydroxyglutaryl-CoA dehydratase (101197, 101198). Also four further proteins were very abundant in acetone-grown CFE: a 2-oxoglutarate ferredoxin oxidoreductase subunit (101176), an acetyl-CoA carboxylase, carboxyltransferase (101177), a long-chain acyl-CoA synthetase (101178) and an acetyl-CoA C-acetyltransferase (101180). The genes of these four proteins are directly adjacent to each other which might imply a potential complex formation. Additionally, proteins annotated as a hypothetical protein (101190), an acetyl-CoA C-acetyltransferase (101191), a 2-(1,2-epoxy-1,2-dihydrophenyl)acetyl-CoA isomerase (101193) and a butirosin biosynthesis protein H (101194) were identified to be highly abundant in acetone-grown cells compared to butyrate-grown cells.

**Fig. 4:**
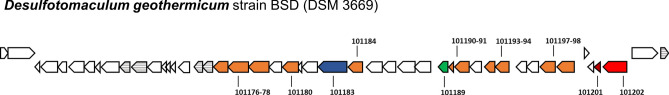
Gene cluster of *D. geothermicum* containing genes coding for proteins relevant for acetone degradation. Color-coded genes are all highly abundant in acetone-grown CFE, in comparison to butyrate-grown CFE. Dark blue: acetolactate synthase (101183); red: small and large subunit of a methylmalonyl-CoA mutase (101201, 101202); green: NAD(P)-dependent dehydrogenase (101189); orange: 2-oxoglutarate ferredoxin oxidoreductase subunit (101176), acetyl-CoA carboxylase (101177), long-chain acyl-CoA synthetase (101178), acetyl-CoA C-acetyltransferase (101180), hydroxymethylglutaryl-CoA lyase (101184), hypothetical protein (101190), acetyl-CoA C-acetyltransferase (101191), 2-(1,2-epoxy-1,2-dihydrophenyl)acetyl-CoA isomerase (101193), Butirosin biosynthesis protein H (101194), and two subunits of a benzoyl-CoA reductase/2-hydroxyglutaryl-CoA dehydratase (101197, 101198); gray-striped genes are constitutively expressed in all conditions; white-labeled genes were not detected in proteome analysis (color figure online)

The genes of all above-mentioned proteins are located in the same gene cluster (see Fig. 4). Furthermore, several other proteins were identified at high abundance in acetone-grown CFE that are located in different gene clusters: a medium-chain acyl-CoA synthetase (107122), an acetyl-CoA synthetase (108124), a FMN-dependent dehydrogenase (10536), and an enoyl-CoA hydratase/carnitine racemase (11458).

### General Genomic and Proteomic Properties

**Fig. 5:**
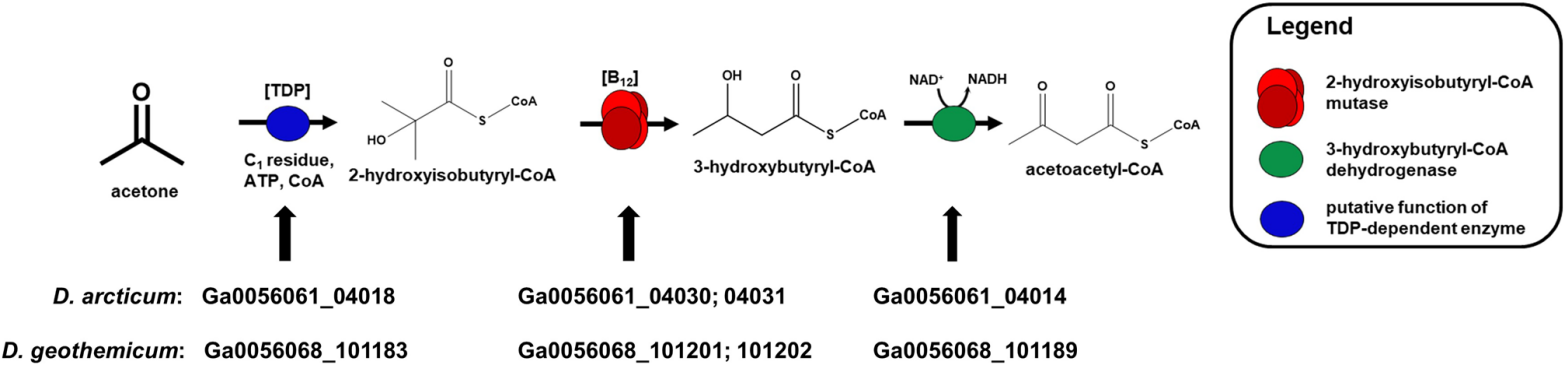
Proposed pathway of acetone degradation including the respective genes. Acetone is activated to 2-hydroxyisobutyryl-CoA by a TDP-dependent enzyme, followed by a B_12_-dependent mutase that linearizes 2-hydroxyisobutyryl-CoA to 3-hydroxybutyryl-CoA which is subsequently oxidized to acetoacetyl-CoA by a dehydrogenase

Genomes of *D. arcticum* and *D. geothermicum* are available at the IMG database of the Joint Genome Institute (JGI IMG) (Fig. 5).

*Desulfotomaculum arcticum* and *Desulfotomaculum geothermicum* both possess a complete set of enzymes for the complete oxidation of acetyl-residues via the reversed Wood–Ljungdahl pathway in their genomes. All respective proteins were identified in all applied growth conditions in both organisms using proteomic analysis. Furthermore, in the genomes of *D. arcticum* and *D. geothermicum* all genes of the tricarboxylic acid (TCA) cycle were found, with the exception of citrate synthase. This gene is missing in both organisms.

Both *Desulfotomaculum* strains are described as sulfate-reducing organisms, therefore, the genomes were examined for respective genes, and expression of these genes was confirmed via proteomic data [18, 19]. Both bacteria harbor genes of a complete pathway for dissimilatory sulfate reduction and also the respective proteins are expressed.

### General Considerations

The two examined *Desulfotomaculum* species *D. arcticum* and *D. geothermicum* both show the ability to grow with acetone as sole carbon source under sulfate-reducing conditions. In general, both employ a similar set of proteins implying a similar degradation pathway. However, as no homolog of the *D. arcticum* protein annotated as threonine dehydrogenase (04035) is found in *D. geothermicum*, this protein appears not to be essential for growth with acetone, even if this protein is highly abundant after growth of *D. arcticum* and of *D. biacutus* with acetone [14]. Comparison of the peptide sequence of the threonine dehydrogenase of *D. arcticum* (Ga0056061_04035) with the respective threonine dehydrogenase of *D. biacutus* (DebiaDRAFT_04514) exhibited an identity of 69.52% at 99% query cover (NCBI Protein Blast). With such a high identity a similar protein function is very likely. The respective protein of *D. biacutus* acted as an oxidoreductase on alcohols and ketones which was proposed to have a detoxifying function by scavenging reactive side products [20].

Moreover, several proteins were found at high abundance in extracts of acetone-grown cells of *D. arcticum* and *D. geothemicum* that are also described as acetone-induced proteins in *D. biacutus*. Of special interest are two subunits of a protein annotated as benzoyl-CoA reductase (DebiaDRAFT_04515, 04516). Similar genes are present also in *D. cetonica* (Ga0122881_11156, 11157) and were identified in both *Desulfotomaculum* strains (see above). Furthermore, a protein annotated as hydroxymethylglutaryl-CoA lyase (HMGL) is again very abundant and acetone-specific in both examined strains. It is also found in proteomic data of *D. biacutus* (DebiaDRAFT_00007; originally annotated as isopropylmalate/homocitrate/citramalate synthase) and is also present in the genome of *D. cetonica* (Ga0122881_105210) directly next to the proposed acetone-degrading genes. The identity at the amino acid level is quite high with 44.72% for the respective protein of *D. geothermicum*, 46.41% for *D. arcticum,* and 88.35% for *D. cetonica* if compared to *D. biacutus*. The amino acid identity of the respective proteins of *D. arcticum* and *D. geothermicum* to each other is at 89.02% (using NCBI BlastP tool). As these proteins are present in all four strains, one or more of these proteins are likely to form a complex which contributes to the activation of acetone.

Moreover, several proteins like acetyl-CoA carboxylases/carboxyltransferases, acyl-CoA synthetases and acetyl-CoA acetyltransferases have been identified to be acetone-specific in both *Desulfotomaculum* strains and were described to be acetone-induced in *D. biacutus* as well [14]. It is very likely that these proteins may be involved in the formation of an activated formyl residue (e.g., formyl-CoA).

Interestingly, in acetone- and butanone-grown cells of *D. arcticum* a hypothetical protein (04016) was identified that is directly adjacent to the TDP-dependent enzyme. Additionally, in acetone-grown cells a pyruvate-formate lyase-activating enzyme was present (but with low coverage) which is also in direct neighborhood to the TDP-dependent enzyme. It seems possible that these two proteins might be needed as subunits or activating enzymes for the production of a functional TDP enzyme.

Including the two *Desulfotomaculum* species described in the present study, there are now four described acetone-degrading, sulfate-reducing bacteria. All four bacteria appear to employ the same pathway for acetone degradation and are known as complete oxidizers that employ the reversed Wood–Ljungdahl pathway for oxidation of acetyl-residues [18, 19, 21, 22]. One might speculate about a possible involvement of some of these enzymes in the formation of an activated formyl residue (e.g., as enzyme-bound carbon monoxide, or as formyl tetrahydrofolate).
